# Altered Fronto-Temporal Functional Connectivity in Individuals at Ultra-High-Risk of Developing Psychosis

**DOI:** 10.1371/journal.pone.0135347

**Published:** 2015-08-12

**Authors:** Youngwoo Bryan Yoon, Je-Yeon Yun, Wi Hoon Jung, Kang Ik K. Cho, Sung Nyun Kim, Tae Young Lee, Hye Yoon Park, Jun Soo Kwon

**Affiliations:** 1 Department of Brain and Cognitive Sciences, College of Natural Sciences, Seoul National University, Seoul, Republic of Korea; 2 Department of Psychiatry, Seoul National University College of Medicine, Seoul, Republic of Korea; 3 Department of Psychology, University of Pennsylvania, Philadelphia, Pennsylvania, United States of America; 4 Institute of Human Behavioral Medicine, Seoul National University-Medical Research Center, Seoul, Republic of Korea; Vanderbilt University, UNITED STATES

## Abstract

**Background:**

The superior temporal gyrus (STG) is one of the key regions implicated in psychosis, given that abnormalities in this region are associated with an increased risk of conversion from an at-risk mental state to psychosis. However, inconsistent results regarding the functional connectivity strength of the STG have been reported, and the regional heterogeneous characteristics of the STG should be considered.

**Methods:**

To investigate the distinctive functional connection of each subregion in the STG, we parcellated the STG of each hemisphere into three regions: the planum temporale, Heschl’s gyrus, and planum polare. Resting-state functional magnetic resonance imaging was obtained from 22 first-episode psychosis (FEP) patients, 41 individuals at ultra-high-risk for psychosis (UHR), and 47 demographically matched healthy controls.

**Results:**

Significant group differences (in seed-based connectivity) were demonstrated in the left planum temporale and from both the right and left Heschl’s gyrus seeds. From the left planum temporale seed, the FEP and UHR groups exhibited increased connectivity to the bilateral dorsolateral prefrontal cortex. In contrast, the FEP and UHR groups demonstrated decreased connectivity from the bilateral Heschl’s gyrus seeds to the dorsal anterior cingulate cortex. The enhanced connectivity between the left planum temporale and right dorsolateral prefrontal cortex was positively correlated with positive symptom severity in individuals at UHR (*r* = .34, *p* = .03).

**Conclusions:**

These findings corroborate the fronto-temporal connectivity disruption hypothesis in schizophrenia by providing evidence supporting the altered fronto-temporal intrinsic functional connection at earlier stages of psychosis. Our data indicate that subregion-specific aberrant fronto-temporal interactions exist in the STG at the early stage of psychosis, thus suggesting that these aberrancies are the neural underpinning of proneness to psychosis.

## Introduction

The superior temporal gyrus (STG) plays an important role in auditory perception and cognitive functions, including language [1], and impairment in this area has been identified as the core pathophysiology of psychosis [2, 3]. Patients with schizophrenia and first-episode psychosis (FEP) consistently exhibit decreased gray matter (GM) volume in the STG [4, 5] and abnormal language task-related functional connectivity networks within the STG [6–8]. Even before the onset of psychosis, an aberrant connection between the STG and prefrontal cortex was noted in individuals with an at-risk mental state (ARMS) during working memory tasks [8]. Furthermore, the degree to which of the GM volume is reduced in the STG is associated with the onset of psychosis in ARMS individuals [9, 10]. These studies suggest that aberrations of the STG may be related to the pathological process of psychosis.

Previous studies have reported discrete patterns in the cytoarchitectonic organization [11, 12] and enzyme distribution [13] in the STG. They have also suggested that subregional differences in the architectonic arrangements influence its connectivity pattern to other areas of the brain [14, 15]. The STG consists of at least three histologically distinctive areas [16], and it can be anatomically divided into three different subregions [17] that exhibit different roles: the planum temporale (PT), Heschl’s gyrus (HG), and the planum polare (PP). Although few studies have carefully explored the various STG subregions, a volumetric study investigated progressive GM loss in the STG subregions in patients transitioning into psychosis. The study demonstrated a distinct pattern for each of the subregions and suggested the importance of assessing the STG by using a more detailed approach [18]. A functional anatomy study suggested that the STG subregions are projected to different parts of the brain and that these projections are involved in different language processing streams [19]. Meaningful studies have investigated the functional connection of the STG subregions in schizophrenia patients, further linking the aberrant connectivity to hallucinations. When comparing patients with and without auditory hallucinations, the aberrant functional connectivity of HG, increased connections to the frontoparietal region and decreased connections to the hippocampus and thalamus were reported among patients with auditory hallucinations [20]. A reduction in the functional connection of the PT with the temporal, parietal, and limbic regions was observed in schizophrenia patients and their relatives, which correlated with the tendency to experience hallucinations [21]. However, no study has revealed how each subregion is distinctively connected to other areas in the brain or how those connections interact with each other.

Whereas task-dependent functional network analyses could underestimate the networks not relevant to the task-dependent circuits, task-independent functional network analyses can identify submerged neural networks rather than an induced phenomenon, which enables us to understand the comprehensive properties of brain physiology [22]. Recently, instead of measuring functional networks across tasks, an alternative approach for measuring the spontaneous dynamics of the brain involves functional magnetic resonance imaging (fMRI) during the resting state [23]. Although few studies have investigated the resting-state network of the STG, moderate evidence suggests network alterations in the region. Some studies have directly explored the resting-state networks of the auditory perception areas in schizophrenia patients [20, 21, 24–26], and other studies have indirectly identified the networks within the auditory-associated area that are connected to other regions of the brain [27, 28]. However, there is inconsistency in the reports regarding the increased or decreased strength of connectivity within the auditory perception areas due to the non-homogeneous characteristics of the subregions in the temporal lobe [27].

We hypothesized that the discrepancy regarding the resting-state functional connectivity strength of the STG is caused by the different characteristics of each subregion. Given the advantages of resting-state studies and the distinctive role of STG subregional networks in individuals at an ultra-high-risk of developing psychosis, we compared the resting-state functional connectivity of the three subregions of the STG to the entire brain using the parcellated seeds of the STG according to the Destrieux atlas [17]. This led to the hypothesis that alterations in functional networks within the STG are closely related to psychosis progression; thus, the networks may demonstrate distinctive patterns in individuals at ultra-high-risk for psychosis (UHR) and in patients shortly after the onset of psychosis. In addition, the alteration is potentially associated with symptom severity. To directly examine the functional connectivity pattern of the entire brain with the STG, voxel-wise seed-based correlation analyses were applied during the resting-state by using different parts of the STG as seeds. Then, we extended our findings to characterize the circuit-level alterations associated with psychotic symptom severity. We will further explore how the presence of hallucinations is reflected in the altered connections. We expect our approach to unearth buried networks that were concealed by other task-dependent networks and monolithically defined seeds.

## Materials and Methods

### Participants

Twenty-four FEP patients, forty-two UHR individuals, and forty-eight healthy controls (HC) were selected. The FEP and UHR subjects were recruited from a longitudinal project to study individuals at a high risk for developing psychosis at the Seoul Youth Clinic, Seoul, Republic of Korea [29, 30]. For the present study, individuals were selected from the subject pool of the previous forty-one months (April 2010 to August 2013). The participants initially contacted the Seoul Youth Clinic via our website, telephone or referral from local clinics.

All participants were interviewed based on the Structured Clinical Interview for DSM-IV (SCID) Axis I [31] by experienced psychiatrists. The Positive and Negative Syndrome Scale (PANSS) [32] was applied to the FEP and UHR groups, and individuals scoring equal to or greater than 3 in the hallucinatory behavior subscale were considered to have hallucinations. The Korean version of the Wechsler Adult Intelligence Scale [33] was administered to all participants to assess their intelligence quotient (IQ).

The inclusion criteria for FEP were defined as individuals satisfying the diagnosis of brief psychotic disorder, schizophreniform disorder, schizophrenia or schizoaffective disorder according to the DSM-IV criteria and exhibiting symptoms for less than 1 year. All UHR subjects were assessed according to the Structured Interview for Prodromal Syndromes [34]. Our UHR subjects satisfied at least one of the prodromal states of psychosis criteria: attenuated positive syndrome (APS), brief intermittent psychotic syndrome (BIPS), and/or genetic risk with deterioration (GRD). APS is defined as the presence of any positive items on the Scale of Prodromal Symptoms (SOPS) [35] in the prodromal range, with an occurrence of the symptoms within the past year, or exhibiting attenuated psychotic symptoms upon at least one point within the past year and showing these symptoms at one or more occasions per week for the past month. BIPS subjects exhibited at least one symptoms from the positive items on the SOPS scale in the psychotic range, with symptoms initiated within the past 3 months and the presence of the symptoms several minutes a day at least once per month. GRD is defined as a significant decline in functioning, showing at least a 30% decrease in the Global Assessment of Functioning (GAF) scale over the past year, and individuals who have a genetic risk due to a first-degree relative with any psychotic disorder or schizotypal personality disorder [36].

At the time of enrollment, seventeen FEP patients were receiving antipsychotics. These patients were also receiving mood stabilizers (*n* = 1), antidepressants (*n* = 3), and anxiolytics (*n* = 8). None of the UHR subjects were medicated with antipsychotics or mood stabilizers, but they were receiving antidepressants (*n* = 4) and anxiolytics (*n* = 4).

HC subjects were recruited from internet advertisements. All HC were screened and confirmed using the SCID Non-patient Edition [37]. None of the HC had any history of a psychiatric disorder or any first- to third-degree biological relatives diagnosed with a psychiatric disorder. The participants were excluded if they were considered to have any of the following: i) a known history of a psychotic disorder, neurological illness, substance abuse, or considerable head injury; ii) evidence of a medical disease with documented cognitive sequelae; or iii) an intellectual disability (IQ below 70).

The present study was performed in accordance with the Declaration of Helsinki. This study was approved from the Institutional Review Board of Seoul National University Hospital. After providing a complete description of the study to the participants, written informed consent was obtained from each participant before study inclusion. For minors who were enrolled in this study, written informed consent was obtained from both the participants themselves and their caretakers or guardians.

### Image Acquisition and Data Preprocessing

Functional and structural images were obtained with a Siemens 3T Trio MRI scanner (Siemens Magnetom Trio, Erlangen, Germany) using a 12-channel head coil. The T1-weighted anatomical image was acquired using magnetization prepared rapid gradient echo (echo time [TE] / repetition time [TR] = 1.89 / 1670 ms, field of view [FOV] = 250 mm, flip angle = 9°, matrix = 256 × 256, voxel size = 1.0 × 1.0 × 1.0 mm^3^, 208 slices). For each subject, we collected a rest scan comprising 116 contiguous echo-planar imaging (EPI) functional images (TE / TR = 30 / 3500 ms, FOV = 240 mm, flip angle = 90°, matrix = 128 × 128, voxel size = 1.9 × 1.9 × 3.5 mm^3^, 35 slices). During resting-state image acquisition, the participants were asked to relax with their eyes closed. To limit possible head movements and subsequent motion artifacts, cushions were used, and the participants were instructed to move as little as possible. The time required to collect the resting-state scans was 6 minutes and 58 seconds.

The first four echo-planar images were discarded. The remaining 112 contiguous EPI functional volumes were preprocessed using the Statistical Parametric Mapping software package, version 8 (SPM8; www.fil.ion.ucl.ac.uk/spm; Wellcome Department of Cognitive Neurology, London, UK). The images were first processed by slice-timing correction and subsequently realigned to correct for head motions. Two subjects from the FEP group, one subject from the UHR group and one subject from the HC group were excluded for exceeding the head motion criteria, i.e., translation > 2.5 mm and rotation > 2.5° in any directions. The functional volumes were then co-registered to each participant's structural volumes. The images were segmented into GM, white matter (WM), and cerebrospinal fluid (CSF) partitions and were spatially normalized to the Montreal Neurological Institute (MNI) standardized space (http://www.mni.mcgill.ca/). The functional volumes were resampled to a 3 × 3 × 3 mm^3^ voxel size and spatially smoothed with a 6-mm full-width half-maximum (FWHM) isotropic Gaussian kernel.

The CONN-fMRI functional connectivity toolbox (V 14f, http://www.nitrc.org/projects/conn/) was used to remove confounding effects and for further analysis. For the nuisance regression, the head motion measured in 6 dimensions with their first derivatives, and the component-based noise correction (CompCor) [38] noise components were designated as nuisance variables. The CompCor strategy built in the CONN toolbox was used to increase the precision of the GM signal by erasing physiological noise, such as the heart rate and respiration signals, and to remove principal components from both the WM and CSF signals. In addition, the CompCor strategy is reported to remove motion-related artifacts effectively [39]. Subsequently, the linear trend was removed through the time course, and the band-pass filter (.008 < *f* < .09 Hz) was applied.

### Functional Connectivity Analysis

We measured the connectivity patterns of the regional mean time series in the resting-state fMRI data using six different seed regions-of-interests (ROI) located in the STG. Freesurfer image analysis (http://surfer.nmr.mgh.harvard.edu/) was used to parcellate the STG into six different ROIs in both hemispheres: HG, PT, and PP (S1 Fig). The six different ROIs of the STG were defined using the Destrieux atlas from the Freesurfer image analysis suite; thus, the seed locations were independent of our data [17].

The functional connectivity networks were measured in the EPI time series. Pearson’s correlation (bivariate) analyses were performed to estimate the connection from the seed ROIs to other voxels in the entire brain. By setting the explicit mask images, between-group comparisons could be performed among voxels presenting significant connectivity with each seed ROI. The explicit mask images were created by applying a cluster-level family-wise error (FWE) rate of *p* < .05 to correct for multiple comparisons over the entire brain. Then, three mask images from each group were combined; thus, all of the significant voxels of the 3 groups were included in a single explicit mask. The combined group explicit masks were applied to prevent potential bias by using the mask acquired from individuals in the psychosis spectrum or the healthy controls alone.

Significant between-group differences were compared by entering the (Fisher’s Z transformed) connectivity map of each ROI into a one-way analysis of variance (ANOVA) model. Regions of significant difference were defined by the clusters surviving the voxel-level height threshold of uncorrected *p* < .001 and the cluster-level extent threshold of *p* < .05 that was corrected for multiple comparisons using the FWE rate. For the post-hoc tests and correlation analyses, the Region of Interest Extraction Tool (http://software.incf.org/software/rex) in the CONN toolbox was used to extract Fisher's Z transformed signal intensity values of the selected clusters. The post-hoc tests were executed using the Bonferroni correction. Correlation analyses were performed to explore the relationship among different functional connectivities and to measure the correlation between an individual’s connectivity intensities and clinical symptom scores from the PANSS. In addition, a two sample t-test was performed on the connectivity strength of individuals with and without hallucinations to compare the connectivity strength between each group.

## Results

### Demographic and Clinical Characteristics

No significant differences in age, gender, education level, handedness, or IQ were noted. The FEP group [duration of untreated psychosis: 0.54 ± 0.32 years] exhibited an increased total score (*p* = .04) and positive score (*p* = .03) in the PANSS scale, which indicated that these patients displayed worse symptom severity compared with the UHR group [duration of untreated prodromal positive symptoms [40]: 1.70 ± 1.49 years]. There were no significant differences in the hallucinatory behavior subscale in the PANSS in both the FEP and UHR groups (*p* = .05). Eleven of the 22 individuals from the FEP group and 12 of the 41 participants from the UHR group scored greater than or equal to 3 on the hallucinatory behavior subscale in the PANSS. The demographical and clinical characteristics are summarized (Table 1).

**Table 1:** Demographic and Clinical Characteristics.

	FEP		UHR		HC		Statistics	
	(*n* = 22)		(*n* = 41)		(*n* = 47)		*F* / χ2	*p*
	Mean	SD	Mean	SD	Mean	SD		
Age (year)	22.73	4.89	20.78	2.52	22.09	2.96	2.995	.054
Gender (M / F)	9 / 13		29 / 12		28 / 19		5.312	.070
Education (year)	13.32	1.94	13.17	1.22	13.94	1.55	3.010	.053
Handedness (R / L)	20 / 2		37 / 4		43 / 4		.041	.980
Estimated IQ	104.59	11.38	111.32	12.14	110.66	11.12	2.711	.071
Clinical rating scales	Mean	SD	Mean	SD			*t*	*p*
PANSS								
Total	68.09	10.41	61.27	13.19			-2.099	.040
Positive	15.45	4.51	12.95	2.81			-2.368	.025
Negative	17.41	4.62	16.24	5.66			-.828	.411
General	35.23	5.90	32.07	7.70			-1.673	.099
GAF	48.36	8.77	50.51	7.56			1.017	.313

*Note*: FEP, first-episode psychosis; UHR, ultra-high-risk for psychosis; HC, healthy control; IQ, Intelligence quotient. PANSS, Positive and Negative Syndrome Scale; GAF, Global Assessment of Functioning

### Functional Connectivity Analysis

**Fig 1:**
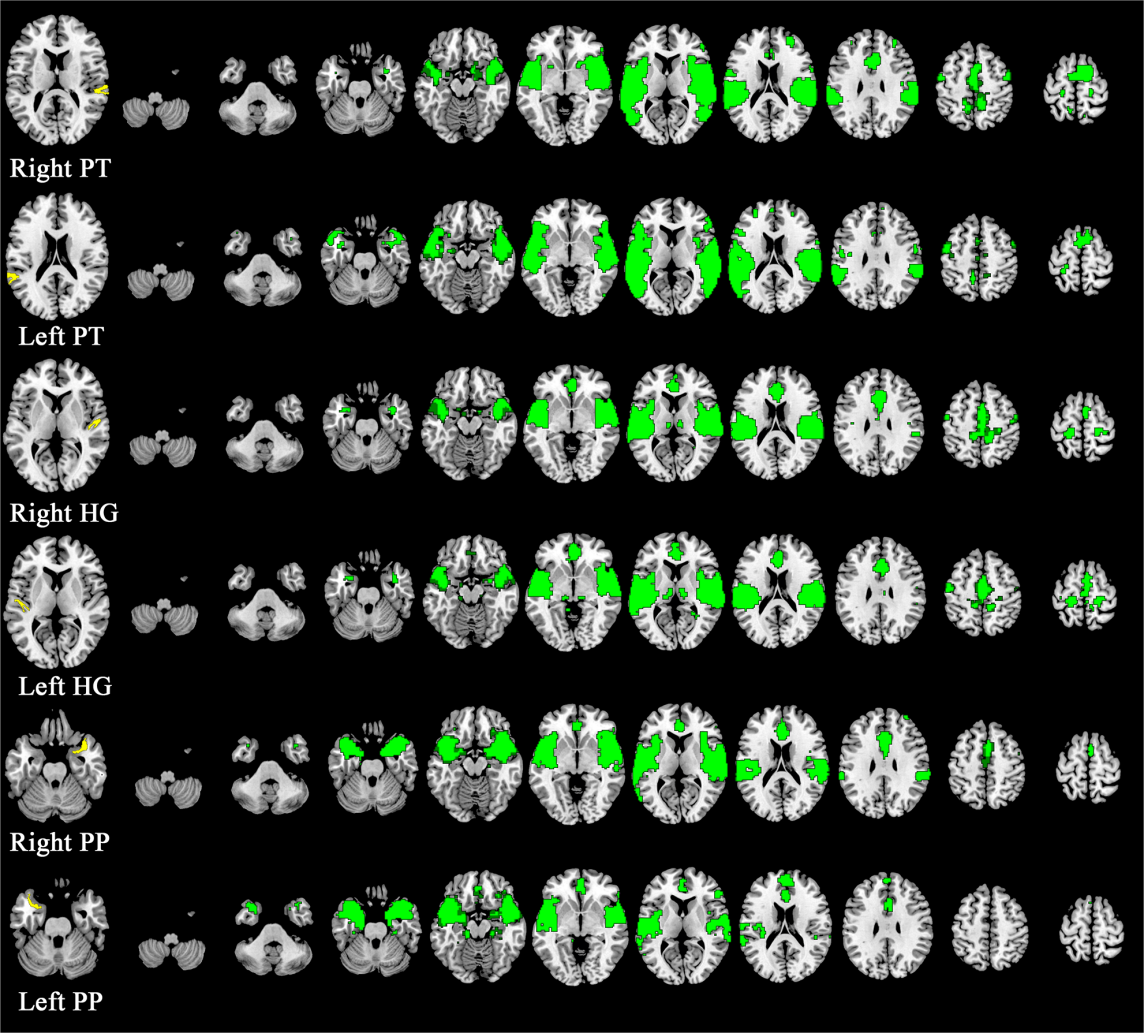
One sample t-tests in healthy controls (HC) to demonstrate the functional connectivity of each subregion of the STG. The far left illustrates the location of each seed: right planum temporale (PT), left PT, right Heschl’s gyrus (HG), left HG, right planum polare (PP), and left PP in yellow (from top to bottom). The green color indicates a significant functional connectivity map of the HC. Axial slices are presented at *z* = -50, -38, -26, -15, -4, 8, 19, 30, 42, 53, and 64 (from left to right) (displayed at *p* < .05, family-wise error rate corrected).

**Fig 2:**
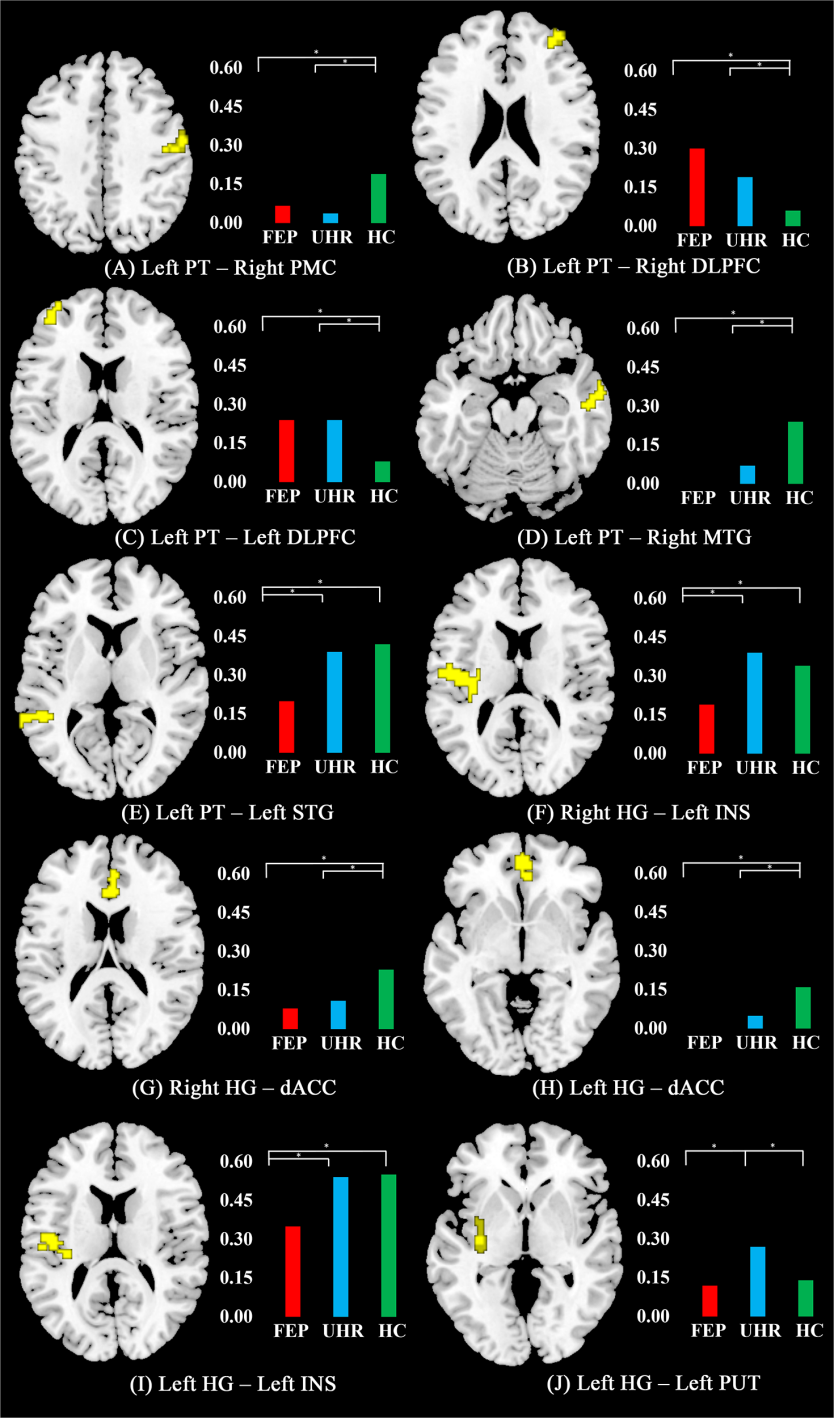
Significant difference in functional connectivity among groups. The differences among individuals with first-episode psychosis (FEP), ultra-high-risk for psychosis (UHR), and the healthy controls (HC) were revealed by one-way ANOVA (*p* < .05, family-wise error rate corrected). Significant differences (presented in yellow) were revealed between the (A) left planum temporale (PT) and right primary motor cortex (PMC); (B) left PT and right dorsolateral prefrontal cortex (DLPFC); (C) left PT and left DLPFC; (D) left PT and right middle temporal gyrus (MTG); (E) left PT and left superior temporal gyrus (STG); (F) right Heschl’s gyrus (HG) and left insular cortex (INS); (G) right HG and dorsal anterior cingulate cortex (dACC); (H) leftt HG and dACC; (I) left HG and left INS; (J) left HG and left putamen (PUT). The functional connectivity coefficients (mean value) of each region showing significant spatial differences are presented in a bar graph.

Each seed region exhibited distinct functional connectivity patterns (Fig 1). The significant spatial group differences of the three groups revealed by the one-way ANOVA model were observed in the left PT and in the right and left HG seed-to-brain networks. Five regions from the left PT seed, 2 regions from the right HG seed, and 3 regions from the left HG seed demonstrated significant group differences. The UHR group demonstrated an intermediate strength between the FEP and HC groups in the 7 of the 10 altered functional connectivities. No statistical significance was observed for the seed-to-brain networks from the right PT as well as the right and left PP seeds. Information regarding the clusters demonstrating significant differences and the post-hoc analysis results is summarized (Table 2 and Fig 2).

**Table 2:** Between Group Difference in Functional Connectivity of Superior Temporal Gyrus.

Seed Region	Brain Region	MNI Coordinate	Size of Clusters	Cluster *p* value	Group	Functional Connectivity		FEP-HC		UHR-HC		FEP-UHR
		(*x*, *y*, *z*)	(# of Voxels)	(FWE corrected)		Mean	SD					
Left planum temporale	Right Primary Motor Cortex	+58–04 +34	165	.009	FEP	0.07	0.12	.007	< .001		1.000	
					UHR	0.04	0.15					
					HC	0.19	0.16					
	Right Dorsolateral prefrontal cortex	+40 +52 +24	138	.021	FEP	0.30	0.15	< .001	.001		.061	
					UHR	0.19	0.16					
					HC	0.06	0.18					
	Left Dorsolateral prefrontal cortex	-38 +50 +22	135	.023	FEP	0.24	0.16	< .001	< .001		1.000	
					UHR	0.24	0.15					
					HC	0.08	0.14					
	Right Middle temporal gyrus	+62 +02–20	135	.023	FEP	0.00	0.19	< .001	< .001		.702	
					UHR	0.07	0.20					
					HC	0.24	0.19					
	Left Superior temporal gyrus	-48–44 +06	119	.040	FEP	0.20	0.16	< .001	1.000		< .001	
					UHR	0.39	0.18					
					HC	0.42	0.15					
Right Heschl's gyrus	Left Insular cortex	-32–20–06	731	< .001	FEP	0.19	0.10	< .001	.179		< .001	
					UHR	0.39	0.12					
					HC	0.34	0.09					
	Dorsal anterior cingulate cortex	0 +28 +18	136	.017	FEP	0.08	0.15	< .001	< .001		1.000	
					UHR	0.11	0.14					
					HC	0.23	0.14					
Left Heschl's gyrus	Dorsal anterior cingulate cortex	0 +50–06	294	< .001	FEP	0.00	0.15	< .001	< .001		.542	
					UHR	0.05	0.12					
					HC	0.16	0.12					
	Left Insular cortex	-44–14 +12	170	.006	FEP	0.35	0.16	< .001	1.000		< .001	
					UHR	0.54	0.13					
					HC	0.55	0.15					
	Left Putamen	-32–16–02	151	.011	FEP	0.12	0.12	1.000	< .001		< .001	
					UHR	0.27	0.11					
					HC	0.14	0.10					

*Note*: FWE, family-wise error; FC, functional connectivity; FEP, first-episode psychosis; UHR, ultra-high-risk for psychosis; HC, healthy control

### Relationship among the Functional Connectivities

In the FEP group, the increased connections between the left PT and the right dorsolateral prefrontal cortex (DLPFC) and decreased connections between the left HG and the dorsal anterior cingulate cortex (ACC) were negatively correlated with each other (*r* = -.48, *p* = .02).

### Association between the Functional Connectivities and Symptom Severity

**Fig 3:**
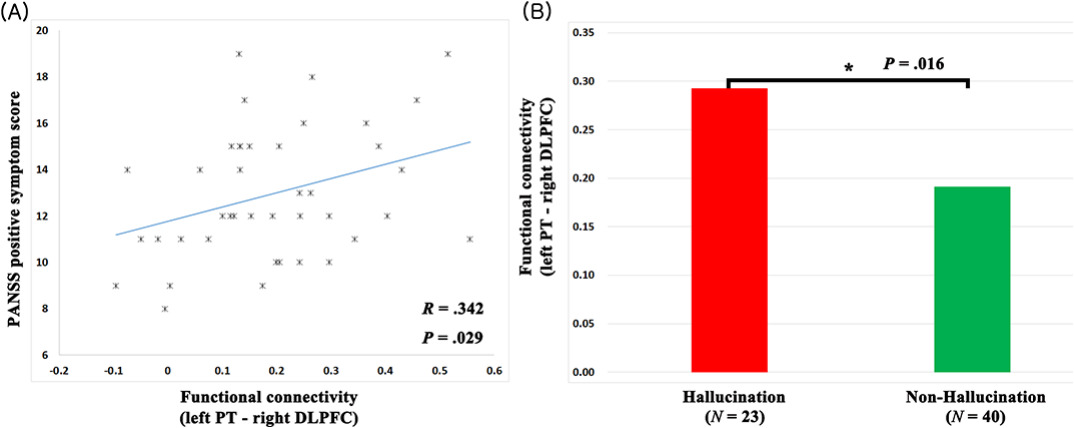
Associations between functional connectivity strength and symptom severity. (A) Scatterplot of the correlation between the overall positive symptom severity and altered functional connectivity of the ultra-high-risk for psychosis (UHR) subject group (*r* = .34, *p* = .03): Fisher's Z transformed connectivity strength between the left planum temporale (PT)–right dorsolateral prefrontal cortex (DLPFC) (*x* axis) and the overall positive symptom scale score of Positive and Negative Syndrome Scale (PANSS) (*y* axis). (B) Bar plot of the mean functional connectivity strength (Fisher's Z transformed) of the hallucination group (*n* = 23) and non-hallucination group (*n* = 40) among individuals in the FEP and UHR groups (*p* = .02).

In the UHR individuals, the level of increased connection strength between the left PT seed and the right DLPFC was positively associated with overall positive symptom severity as assessed by the PANSS (*r* = .34, *p* = .03) (Fig 3A). The enhanced connectivity in the UHR participants was positively correlated with the hallucinatory behavior subscale in the PANSS (*r* = .33, *p* = .03). Among individuals in the FEP and UHR groups, there were significant differences in the degree of the augmented connection between individuals with (*n* = 23) and without auditory hallucination (*n* = 40) (*p* = .02) (Fig 3B). None of aberrant connectivity showed a significant association with the lengths of the prodromal syndrome in the UHR group.

## Discussion

This study investigated the resting-state functional connectivity network within the subregions of the STG in FEP and UHR individuals to explore subregion-specific alterations in connectivity. The widespread disorganization of networks, including the frontal area, as measured by the aberrant strength of the functional connectivity, was evident in both the FEP and UHR groups with the left PT and in the right and left HGs. Notably, the strength of the altered network between the left PT and the right DLPFC in the UHR group correlated with psychotic symptom severity. To our knowledge, the present study is the first to report the disorganization in fronto-temporal functional connectivity in both FEP and UHR individuals during the resting-state. Taken together, our results suggest that the alteration in fronto-temporal functional connectivity is evident at the earliest stages and the prodromal stages of psychosis.

The hyperconnectivity between the left PT and the bilateral DLPFC and the hypoconnectivity between the bilateral HG and the dorsal ACC demonstrate the existence of disorganized functional connections between the prefrontal cortex and the STG. The dysconnection hypothesis of schizophrenia comprises not only hypoconnectivity, which refers to decreased interactions between regions, but also hyperconnectivity, which represents reinforcement between regions [41–43]. Previously, both hyper- and hypoconnectivity in fronto-temporal functional connectivity have been reported [6, 24, 27, 44, 45], and our results demonstrate the distinctive fronto-temporal connectivity patterns in different subregions of the STG and are thus consistent with previous findings. No significant difference in fronto-temporal connectivity strength was noted between the FEP and UHR groups; however, all of the altered fronto-temporal connections demonstrated the highest degrees of dysconnectivity in the FEP individuals, and intermediate values were noted in the UHR group. These results resemble previous studies investigating WM disconnections [46] and fronto-temporal functional connectivity during working memory tasks in UHR and FEP subjects [8].

The hyperconnectivity between the left PT and the right DLPFC was positively correlated with the overall positive symptom severity, as measured by the PANSS, among the UHR individuals (Fig 3A). The relationship between the disorganization in neural activity and the positive symptoms has been discussed for decades [47]. In particular, the hyperconnectivity of the neural circuitry is thought to be due to excessive attention to inconsequential information [48]. We suggest that the excessive attention induces positive symptoms, which are consequences of impairment in selective attention [49]. Additionally, the relationship between hyperconnectivity and hallucinatory symptom severity was analyzed, and a positive correlation among UHR individuals was observed. There were significant differences in the degree of hyperconnectivity between participants with or without hallucinatory symptoms among the FEP and UHR groups (Fig 3B). A meta-analysis revealed that the core regions responsible for hallucinations are the regions related to speech production, whereas an individual’s hallucinatory trait is associated with the speech perception areas [50]. The DLPFC is involved in numerous high-level functions, including speech perception [51]. Collectively, an enhanced connection between both speech perception regions leads to an exaggerated attention to irrelevant cues, which contributes to an individual’s proneness to hallucinations. However, we were unable to reveal an association between the positive symptom severity and aberrant fronto-temporal connectivity in the FEP group. The differences in fronto-temporal connections between the FEP and HC groups were greater than those between the UHR and HC groups. We hypothesize that the more severely altered connections or medications in FEP individuals influenced the positive symptoms in a more complex manner; thus, the relationship between a single aberrant connection and positive symptom severity was potentially blurred.

A divergent trend was observed between the left PT to bilateral DLPFC and the bilateral HG to dorsal ACC networks. In addition, the FEP group exhibited decreased connectivity to the insula via the bilateral HG seeds. The ACC and the insula comprises the salience network [52]. The reduced functional connection in the salience network are related to the information processing disturbance in schizophrenia and are regarded as the core pathology of the disease [53, 54]. One possible explanation for the dissimilar trend in fronto-temporal functional connections could be attributed to our brain’s compensatory mechanism when the reduced functional connectivity between the HG and salience network triggers an abnormal increase in top-down control. Evidence supporting this notion was evident in the FEP group, where a negative correlation between the left PT to right DLPFC and the left HG to dorsal ACC connections was noted. The interaction between the auditory cortex and subregions of the salience network were also assessed by electrophysiological studies analyzing mismatch negativity (MMN) [55–58], which is considered a potent biomarker of psychosis [59]. The present findings revealed a decreased interaction between the auditory cortex and the salience network and thus extend our previous electrophysiological studies, which demonstrated a decreased MMN in UHR individuals [60, 61].

There are meaningful studies reporting aberrant connectivity between the prefrontal area and temporal area during various tasks [8, 44, 62]. We expand these task-induced network studies by demonstrating aberrant connections between the prefrontal and temporal areas during the resting-state. Resting-state research is expected to better reflect the trait of brain physiology by presenting comprehensive fluctuations in intrinsic brain activity [63]. Therefore, our findings demonstrate an alteration in the spontaneous neural activity rather than evoked disorganization. Furthermore, the resting-state better represents the phenomenology of schizophrenia compared with the task-induced neural activity [64]. Because our findings were obtained from FEP and UHR individuals, we suggest that the aberrant connectivity, which has been consistently reported between the temporal and frontal lobes in schizophrenia patients, potentially existed from earlier stages of the illness. Our results conclusively show that the alteration in fronto-temporal functional connectivity is a trait noted in individuals at early or prodromal psychosis and that the aberrant connection is rather intrinsic. These findings suggest that the functional disjunction is a candidate phenotype for the transition to psychosis.

Our study includes a few limitations. First, although none of our UHR participants were receiving antipsychotics, 17 of our 22 FEP participants were taking antipsychotics. However, we did not observe a significant difference in connection strength in the disease-affected connections between FEP patients taking antipsychotics and FEP patients who were not administered antipsychotics. Regardless, the effect of medication on connectivity should be investigated in further studies. Second, compared with our UHR and HC groups, a relatively smaller number of FEP individuals were included in our study. No significant group differences in the right PT and bilateral PP seeds were noted, and the between-group differences of these seed-based connections may be underestimated due to the small size of the FEP group. Third, an association between functional connectivity strength and symptom severity was only noted in the UHR group. Our UHR group exhibited significantly milder positive symptom severity compared with the FEP group. This fact could potentially serve as one of the strengths of our study. The UHR group exhibited symptoms at a subthreshold for psychosis, which suggests that the enhancement in connectivity is not the consequence of severe psychotic symptoms. To validate our conclusions, a longitudinal effect of enhanced connections to positive symptoms, including hallucinations, should be verified in a follow-up study.

In summary, our results identified altered temporal and cortical functional connectivity in FEP patients and UHR individuals. Disorganized fronto-temporal connectivity was observed in both groups, and such disorganization was associated with psychotic symptom severity. By highlighting fronto-temporal functional connectivity alterations in early psychosis patients and UHR individuals, our results clarify the controversy regarding hyper- or hypoconnectivity in resting-state fronto-temporal functional connectivity. Furthermore, the aberrant fronto-temporal connections were associated with psychotic symptom severity, thus emphasizing the clinical significance of the circuit in the progression of psychosis. Together with additional studies emphasizing the role of the STG in individuals at ultra-high-risk of developing psychosis, our study extends the previous studies by underscoring the role of the STG as the core hub for functional interactions with other areas of the brain.

## Supporting Information

S1 Fig Placement of primary seed region of interest (ROI). The ROIs are overlaid on a standard neuroanatomical template.
(TIF)

## References

[1] Bigler ED, Mortensen S, Neeley ES, Ozonoff S, Krasny L, Johnson M, et al Superior temporal gyrus, language function, and autism. Dev Neuropsychol. 2007; 31: 217–38. 10.1080/87565640701190841 17488217

[2] Reichenberg A, Caspi A, Harrington H, Houts R, Keefe RS, Murray RM, et al Static and dynamic cognitive deficits in childhood preceding adult schizophrenia: a 30-year study. Am J Psychiatry. 2010; 167: 160–9. 10.1176/appi.ajp.2009.09040574 20048021 PMC3552325

[3] Kahn RS, Keefe RS. Schizophrenia is a cognitive illness: time for a change in focus. JAMA psychiatry. 2013; 70: 1107–12. 10.1001/jamapsychiatry.2013.155 23925787

[4] Barta PE, Pearlson GD, Powers RE, Richards SS, Tune LE. Auditory hallucinations and smaller superior temporal gyral volume in schizophrenia. Am J Psychiatry. 1990; 147: 1457–62. 2221156 10.1176/ajp.147.11.1457

[5] Shenton ME, Kikinis R, Jolesz FA, Pollak SD, LeMay M, Wible CG, et al Abnormalities of the left temporal lobe and thought disorder in schizophrenia. a quantitative magnetic resonance imaging study. N Engl J Med. 1992; 327: 604–12. 10.1056/nejm199208273270905 1640954

[6] Mechelli A, Allen P, Amaro E Jr., Fu CH, Williams SC, Brammer MJ, et al Misattribution of speech and impaired connectivity in patients with auditory verbal hallucinations. Hum Brain Mapp. 2007; 28: 1213–22. 10.1002/hbm.20341 17266108 PMC6871422

[7] Wolf DH, Gur RC, Valdez JN, Loughead J, Elliott MA, Gur RE, et al Alterations of fronto-temporal connectivity during word encoding in schizophrenia. Psychiatry Res. 2007; 154: 221–32. 10.1016/j.pscychresns.2006.11.008 17360163 PMC2359768

[8] Crossley NA, Mechelli A, Fusar-Poli P, Broome MR, Matthiasson P, Johns LC, et al Superior temporal lobe dysfunction and frontotemporal dysconnectivity in subjects at risk of psychosis and in first-episode psychosis. Hum Brain Mapp. 2009; 30: 4129–37. 10.1002/hbm.20834 19530219 PMC6870945

[9] Borgwardt SJ, Riecher-Rossler A, Dazzan P, Chitnis X, Aston J, Drewe M, et al Regional gray matter volume abnormalities in the at risk mental state. Biol Psychiatry. 2007; 61: 1148–56. 10.1016/j.biopsych.2006.08.009 17098213

[10] Fusar-Poli P, Radua J, McGuire P, Borgwardt S. Neuroanatomical maps of psychosis onset: voxel-wise meta-analysis of antipsychotic-naive VBM studies. Schizophr Bull. 2012; 38: 1297–307. 10.1093/schbul/sbr134 22080494 PMC3494061

[11] Galaburda A, Sanides F. Cytoarchitectonic organization of the human auditory cortex. J Comp Neurol. 1980; 190: 597–610. 10.1002/cne.901900312 6771305

[12] Galaburda AM, Pandya DN. The intrinsic architectonic and connectional organization of the superior temporal region of the rhesus monkey. J Comp Neurol. 1983; 221: 169–84. 10.1002/cne.902210206 6655080

[13] Rivier F, Clarke S. Cytochrome oxidase, acetylcholinesterase, and NADPH-diaphorase staining in human supratemporal and insular cortex: evidence for multiple auditory areas. Neuroimage. 1997; 6: 288–304. 10.1006/nimg.1997.0304 9417972

[14] Pandya DN. Anatomy of the auditory cortex. Rev Neurol (Paris). 1995; 151: 486–94. 8578069

[15] Kaas JH, Hackett TA, Tramo MJ. Auditory processing in primate cerebral cortex. Curr Opin Neurobiol. 1999; 9: 164–70. 10322185 10.1016/s0959-4388(99)80022-1

[16] Rajarethinam RP, DeQuardo JR, Nalepa R, Tandon R. Superior temporal gyrus in schizophrenia: a volumetric magnetic resonance imaging study. Schizophr Res. 2000; 41: 303–12. 10708339 10.1016/s0920-9964(99)00083-3

[17] Destrieux C, Fischl B, Dale A, Halgren E. Automatic parcellation of human cortical gyri and sulci using standard anatomical nomenclature. Neuroimage. 2010; 53: 1–15. 10.1016/j.neuroimage.2010.06.010 20547229 PMC2937159

[18] Takahashi T, Wood SJ, Yung AR, Soulsby B, McGorry PD, Suzuki M, et al Progressive gray matter reduction of the superior temporal gyrus during transition to psychosis. Arch Gen Psychiatry. 2009; 66: 366–76. 10.1001/archgenpsychiatry.2009.12 19349306

[19] Hickok G, Poeppel D. Dorsal and ventral streams: a framework for understanding aspects of the functional anatomy of language. Cognition. 2004; 92: 67–99. 10.1016/j.cognition.2003.10.011 15037127

[20] Shinn AK, Baker JT, Cohen BM, Ongur D. Functional connectivity of left Heschl's gyrus in vulnerability to auditory hallucinations in schizophrenia. Schizophr Res. 2013; 143: 260–8. 10.1016/j.schres.2012.11.037 23287311 PMC3601525

[21] Oertel-Knochel V, Knochel C, Matura S, Prvulovic D, Linden DE, van de Ven V. Reduced functional connectivity and asymmetry of the planum temporale in patients with schizophrenia and first-degree relatives. Schizophr Res. 2013; 147: 331–8. 10.1016/j.schres.2013.04.024 23672819

[22] Xiong J, Parsons LM, Gao JH, Fox PT. Interregional connectivity to primary motor cortex revealed using MRI resting state images. Hum Brain Mapp. 1999; 8: 151–6. 10524607 10.1002/(SICI)1097-0193(1999)8:2/3<151::AID-HBM13>3.0.CO;2-5 PMC6873334

[23] Fornito A, Bullmore ET. What can spontaneous fluctuations of the blood oxygenation-level-dependent signal tell us about psychiatric disorders? Curr Opin Psychiatry. 2010; 23: 239–49. 10.1097/YCO.0b013e328337d78d 20216219

[24] Vercammen A, Knegtering H, den Boer JA, Liemburg EJ, Aleman A. Auditory hallucinations in schizophrenia are associated with reduced functional connectivity of the temporo-parietal area. Biol Psychiatry. 2010; 67: 912–8. 10.1016/j.biopsych.2009.11.017 20060103

[25] Gavrilescu M, Rossell S, Stuart GW, Shea TL, Innes-Brown H, Henshall K, et al Reduced connectivity of the auditory cortex in patients with auditory hallucinations: a resting state functional magnetic resonance imaging study. Psychol Med. 2010; 40: 1149–58. 10.1017/s0033291709991632 19891811

[26] Diederen KM, Neggers SF, de Weijer AD, van Lutterveld R, Daalman K, Eickhoff SB, et al Aberrant resting-state connectivity in non-psychotic individuals with auditory hallucinations. Psychol Med. 2013; 43: 1685–96. 10.1017/s0033291712002541 23199762

[27] Zhou Y, Liang M, Jiang T, Tian L, Liu Y, Liu Z, et al Functional dysconnectivity of the dorsolateral prefrontal cortex in first-episode schizophrenia using resting-state fMRI. Neurosci Lett. 2007; 417: 297–302. 10.1016/j.neulet.2007.02.081 17399900

[28] Dandash O, Fornito A, Lee J, Keefe RS, Chee MW, Adcock RA, et al Altered striatal functional connectivity in subjects with an at-risk mental state for psychosis. Schizophr Bull. 2014; 40: 904–13. 10.1093/schbul/sbt093 23861539 PMC4059431

[29] Kwon JS, Byun MS, Lee TY, An SK. Early intervention in psychosis: insights from Korea. Asian J Psychiatr. 2012; 5: 98–105. 26878954 10.1016/j.ajp.2012.02.007

[30] Lee TY, Kim SN, Correll CU, Byun MS, Kim E, Jang JH, et al Symptomatic and functional remission of subjects at clinical high risk for psychosis: a 2-year naturalistic observational study. Schizophr Res. 2014; 156: 266–71. 10.1016/j.schres.2014.04.002 24815568

[31] First MB, Spitzer RL, Gibbon M, Williams JB. Structured Clinical Interview for DSM-IV Patient Edition (SCID-P) New York, NY: Biometrics Research Department, New York State Psychiatric Institute; 1995.

[32] Kay SR, Fiszbein A, Opler LA. The positive and negative syndrome scale (PANSS) for schizophrenia. Schizophr Bull. 1987; 13: 261–76. 3616518 10.1093/schbul/13.2.261

[33] Yum T, Park Y, Oh K, Kim J, Lee Y. The manual of Korean-Wechsler adult intelligence scale Korea Guidance, Seoul 1992.

[34] Miller TJ, McGlashan TH, Rosen JL, Somjee L, Markovich PJ, Stein K, et al Prospective diagnosis of the initial prodrome for schizophrenia based on the Structured Interview for Prodromal Syndromes: preliminary evidence of interrater reliability and predictive validity. Am J Psychiatry. 2002; 159: 863–5. 11986145 10.1176/appi.ajp.159.5.863

[35] Miller TJ, McGlashan TH, Woods SW, Stein K, Driesen N, Corcoran CM, et al Symptom assessment in schizophrenic prodromal states. Psychiatr Q. 1999; 70: 273–87. 10587984 10.1023/a:1022034115078

[36] Jung MH, Jang JH, Kang DH, Choi JS, Shin NY, Kim HS, et al The reliability and validity of the korean version of the structured interview for prodromal syndrome. Psychiatry Investig. 2010; 7: 257–63. 10.4306/pi.2010.7.4.257 21253409 PMC3022312

[37] First MB, Spitzer RL, Gibbon M, Williams JB. Structured Clinical Interview for DSM-IV Axis I Disorders, Non-Patient Edition (SCID-NP) New York, NY: Biometrics Research Department, New York State Psychiatric Institute; 1995.

[38] Behzadi Y, Restom K, Liau J, Liu TT. A component based noise correction method (CompCor) for BOLD and perfusion based fMRI. Neuroimage. 2007; 37: 90–101. 10.1016/j.neuroimage.2007.04.042 17560126 PMC2214855

[39] Muschelli J, Nebel MB, Caffo BS, Barber AD, Pekar JJ, Mostofsky SH. Reduction of motion-related artifacts in resting state fMRI using aCompCor. Neuroimage. 2014; 96: 22–35. 10.1016/j.neuroimage.2014.03.028 24657780 PMC4043948

[40] Fusar-Poli P, Meneghelli A, Valmaggia L, Allen P, Galvan F, McGuire P, et al Duration of untreated prodromal symptoms and 12-month functional outcome of individuals at risk of psychosis. Br J Psychiatry. 2009; 194: 181–2. 10.1192/bjp.bp.107.047951 19182184

[41] Stephan KE, Baldeweg T, Friston KJ. Synaptic plasticity and dysconnection in schizophrenia. Biol Psychiatry. 2006; 59: 929–39. 10.1016/j.biopsych.2005.10.005 16427028

[42] Stephan KE, Friston KJ, Frith CD. Dysconnection in schizophrenia: from abnormal synaptic plasticity to failures of self-monitoring. Schizophr Bull. 2009; 35: 509–27. 10.1093/schbul/sbn176 19155345 PMC2669579

[43] Friston KJ. The disconnection hypothesis. Schizophr Res. 1998; 30: 115–25. 9549774 10.1016/s0920-9964(97)00140-0

[44] Allen P, Stephan KE, Mechelli A, Day F, Ward N, Dalton J, et al Cingulate activity and fronto-temporal connectivity in people with prodromal signs of psychosis. Neuroimage. 2010; 49: 947–55. 10.1016/j.neuroimage.2009.08.038 19703570 PMC3221036

[45] Hoffman RE, Fernandez T, Pittman B, Hampson M. Elevated functional connectivity along a corticostriatal loop and the mechanism of auditory/verbal hallucinations in patients with schizophrenia. Biol Psychiatry. 2011; 69: 407–14. 10.1016/j.biopsych.2010.09.050 21145042 PMC3039042

[46] Carletti F, Woolley JB, Bhattacharyya S, Perez-Iglesias R, Fusar Poli P, Valmaggia L, et al Alterations in white matter evident before the onset of psychosis. Schizophr Bull. 2012; 38: 1170–9. 10.1093/schbul/sbs053 22472474 PMC3494044

[47] Maher BA. Delusional thinking and perceptual disorder. J Individ Psychol. 1974; 30: 98–113. 4857199

[48] Fornito A, Harrison BJ, Goodby E, Dean A, Ooi C, Nathan PJ, et al Functional dysconnectivity of corticostriatal circuitry as a risk phenotype for psychosis. JAMA psychiatry. 2013; 70: 1143–51. 10.1001/jamapsychiatry.2013.1976 24005188

[49] Morris R, Griffiths O, Le Pelley ME, Weickert TW. Attention to irrelevant cues is related to positive symptoms in schizophrenia. Schizophr Bull. 2013; 39: 575–82. 10.1093/schbul/sbr192 22267535 PMC3627774

[50] Kuhn S, Gallinat J. Quantitative meta-analysis on state and trait aspects of auditory verbal hallucinations in schizophrenia. Schizophr Bull. 2012; 38: 779–86. 10.1093/schbul/sbq152 21177743 PMC3406531

[51] Dehaene-Lambertz G, Dehaene S, Hertz-Pannier L. Functional neuroimaging of speech perception in infants. Science. 2002; 298: 2013–5. 10.1126/science.1077066 12471265

[52] Taylor KS, Seminowicz DA, Davis KD. Two systems of resting state connectivity between the insula and cingulate cortex. Hum Brain Mapp. 2009; 30: 2731–45. 10.1002/hbm.20705 19072897 PMC6871122

[53] White TP, Joseph V, Francis ST, Liddle PF. Aberrant salience network (bilateral insula and anterior cingulate cortex) connectivity during information processing in schizophrenia. Schizophr Res. 2010; 123: 105–15. 10.1016/j.schres.2010.07.020 20724114

[54] Palaniyappan L, Liddle PF. Does the salience network play a cardinal role in psychosis? an emerging hypothesis of insular dysfunction. J Psychiatry Neurosci. 2012; 37: 17–27. 10.1503/jpn.100176 21693094 PMC3244495

[55] Waberski TD, Kreitschmann-Andermahr I, Kawohl W, Darvas F, Ryang Y, Rodewald M, et al Spatio-temporal source imaging reveals subcomponents of the human auditory mismatch negativity in the cingulum and right inferior temporal gyrus. Neurosci Lett. 2001; 308: 107–10. 11457571 10.1016/s0304-3940(01)01988-7

[56] Schall U, Johnston P, Todd J, Ward PB, Michie PT. Functional neuroanatomy of auditory mismatch processing: an event-related fMRI study of duration-deviant oddballs. Neuroimage. 2003; 20: 729–36. 10.1016/s1053-8119(03)00398-7 14568447

[57] Gaebler AJ, Mathiak K, Koten JW Jr., Konig AA, Koush Y, Weyer D, et al Auditory mismatch impairments are characterized by core neural dysfunctions in schizophrenia. Brain. 2015; 10.1093/brain/awv049 25743635 PMC5963408

[58] Takahashi H, Rissling AJ, Pascual-Marqui R, Kirihara K, Pela M, Sprock J, et al Neural substrates of normal and impaired preattentive sensory discrimination in large cohorts of nonpsychiatric subjects and schizophrenia patients as indexed by MMN and P3a change detection responses. Neuroimage. 2013; 66: 594–603. 10.1016/j.neuroimage.2012.09.074 23085112 PMC3652903

[59] Belger A, Yucel GH, Donkers FC. In search of psychosis biomarkers in high-risk populations: is the mismatch negativity the one we've been waiting for? Biol Psychiatry. 2012; 71: 94–5. 10.1016/j.biopsych.2011.11.009 22152782

[60] Shin KS, Kim JS, Kim SN, Koh Y, Jang JH, An SK, et al Aberrant auditory processing in schizophrenia and in subjects at ultra-high-risk for psychosis. Schizophr Bull. 2012; 38: 1258–67. 10.1093/schbul/sbr138 22021663 PMC3494059

[61] Shin KS, Kim JS, Kang DH, Koh Y, Choi JS, O'Donnell BF, et al Pre-attentive auditory processing in ultra-high-risk for schizophrenia with magnetoencephalography. Biol Psychiatry. 2009; 65: 1071–8. 10.1016/j.biopsych.2008.12.024 19200950

[62] Benetti S, Pettersson-Yeo W, Allen P, Catani M, Williams S, Barsaglini A, et al Auditory verbal hallucinations and brain dysconnectivity in the perisylvian language network: a multimodal investigation. Schizophr Bull. 2015; 41: 192–200. 10.1093/schbul/sbt172 24361862 PMC4266279

[63] Fox MD, Raichle ME. Spontaneous fluctuations in brain activity observed with functional magnetic resonance imaging. Nat Rev Neurosci. 2007; 8: 700–11. 10.1038/nrn2201 17704812

[64] Malaspina D, Harkavy-Friedman J, Corcoran C, Mujica-Parodi L, Printz D, Gorman JM, et al Resting neural activity distinguishes subgroups of schizophrenia patients. Biol Psychiatry. 2004; 56: 931–7. 10.1016/j.biopsych.2004.09.013 15601602 PMC2993017

