# Supplementary figures and images for: Altered Fronto-Temporal Functional Connectivity in Individuals at Ultra-High-Risk of Developing Psychosis
Source: PLoS One. 2015 Aug 12;10(8):e0135347. doi: 10.1371/journal.pone.0135347 (PMC4534425; doi:10.1371/journal.pone.0135347)

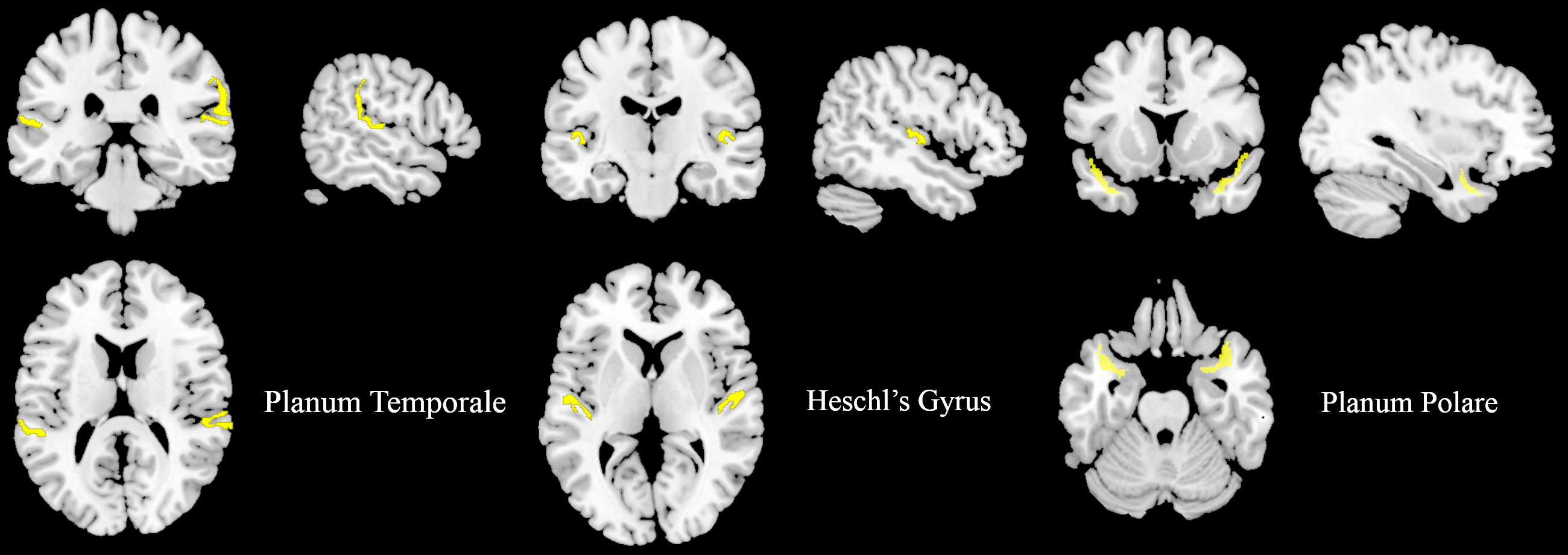

Supplement: S1 Fig — The ROIs are overlaid on a standard neuroanatomical template. (TIF) [file pone.0135347.s001.tif]
